# Cutaneous laser treatment of port-wine stains and its impact on ocular manifestations in Sturge–Weber syndrome

**DOI:** 10.3389/fopht.2026.1843605

**Published:** 2026-06-30

**Authors:** Susan Darby Keith, Grace Herrick, Ashton Arlen, Halrleen K. Multani, Kelly Frasier, Kathryn Dempsey, Paul McDaniel

**Affiliations:** 1 Alabama College of Osteopathic Medicine, Dothan, AL, United States; 2 The University of Texas at Tyler, Tyler, TX, United States; 3 Meharry Medical College School of Medicine, Nashville, TN, United States; 4 Northwell Health, New Hyde Park, NY, United States; 5 Coastal Dermatology & Skin Care Center, Mobile, AL, United States; 6 Southern Eye Group, Mobile, AL, United States

**Keywords:** IPL (Intense Pulsed Light), neurocutaneoous disorder, ocular manifestation, PDT - photodynamic therapy, phakomatoses, port wine stains (PWS), pulsed dye laser (PDL), Sturge-Weber syndrome

## Abstract

Sturge-Weber Syndrome (SWS) is a rare neurocutaneous disorder characterized by the presence of port-wine stains (PWS) and ophthalmologic complications, including glaucoma and choroidal hemangiomas. These manifestations result from somatic mutations in the *GNAQ* gene, leading to vascular malformations that affect both the skin and ocular tissues. This review aims to evaluate the impact of laser treatment for PWS on ocular manifestations in SWS, considering both clinical outcomes and underlying biological mechanisms. PWS are common in SWS patients and significantly impair quality of life (QoL) and necessitate effective treatment strategies. Pulsed dye laser (PDL) therapy, which targets the abnormal blood vessels within PWS, has been established as an effective method for reducing the size and appearance of these stains. Clinical studies suggest that PDL therapy not only improves dermatologic outcomes but may also have possible association with ocular vascular dynamics such as reducing intraocular pressure and ameliorating choroidal hemangiomas. The review evaluates data from various studies and highlights changes in intraocular pressure, the incidence of glaucoma, and modifications in choroidal hemangiomas following laser treatment. Mechanistic insights suggest that laser therapy may improve dermatologic and ocular symptoms by modulating sebaceous gland activity and enhancing the skin’s barrier function, thereby indirectly affecting ocular health. Additionally, the review discusses the safety profiles of different laser systems, the importance of multidisciplinary care, and the need for standardized treatment protocols to minimize risks and optimize patient outcomes. Integrated dermatologic and ophthalmologic care remains crucial in improving the overall health and QoL for patients with SWS.

## 1 Introduction

Sturge-Weber syndrome (SWS) is a rare congenital neurocutaneous disorder classified among the phakomatoses that arises from somatic mosaic mutations in the Guanine Nucleotide-Binding Protein Alpha-q (*GNAQ*) gene (1). The underlying genetic defect, most commonly involving the p.R183Q substitution, disrupts normal vascular development during embryogenesis and contributes to persistent vascular dysregulation (1). SWS affects approximately 1 in 20,000 to 50,000 live births and occurs sporadically without hereditary transmission (2). Clinical manifestations vary considerably in severity and distribution but classically include facial port-wine stains (PWS), leptomeningeal vascular malformations, and ocular abnormalities such as glaucoma and choroidal hemangiomas.

Neurologic complications contribute substantially to morbidity in SWS. Approximately 70% of patients with unilateral leptomeningeal capillary malformations experience seizures, with prevalence increasing to nearly 90% in patients with bilateral involvement during the first two years of life (3). Seizures and associated neurologic dysfunction may contribute to developmental delay and cognitive impairment, particularly in patients with poorly controlled epilepsy (4). However, facial port-wine stains remain the most visible manifestation of Sturge-Weber syndrome and frequently serve as an early clinical marker of disease involvement.

PWS are capillary vascular malformations that initially present as pink or red patches and may progressively darken, thicken, and develop nodularity over time. Beyond cosmetic burden, extensive or periocular PWS may contribute to substantial psychosocial distress and increased risk of ophthalmologic complications (5). PWS involving the palpebral or ophthalmic (V1) trigeminal distribution demonstrate particularly strong associations with glaucoma and choroidal vascular abnormalities (6). Elevated episcleral venous pressure, abnormal ocular vascular development, and congenital anterior chamber abnormalities may contribute to increased intraocular pressure (IOP) and progressive optic nerve injury in affected patients.

Pulsed dye laser (PDL) therapy remains the standard treatment for PWS and functions through selective photothermolysis targeting hemoglobin within abnormal superficial blood vessels. Additional vascular-directed modalities including photodynamic therapy (PDT), intense pulsed light (IPL), and neodymium-doped yttrium aluminum garnet (Nd: YAG) laser therapy have also been explored in selected cases depending on lesion characteristics and treatment response. Importantly, these modalities differ substantially in mechanism of action, tissue penetration, vascular selectivity, efficacy profiles, and adverse effect risk. Although dermatologic improvement following laser therapy is well established, the potential impact of cutaneous vascular treatment on ocular manifestations remains incompletely understood.

Current evidence evaluating the relationship between laser treatment of PWS and ocular outcomes in SWS remains limited and heterogeneous. Existing studies consist primarily of retrospective studies, observational cohorts, case series, and isolated case reports with variable treatment protocols, follow-up duration, and outcome assessment. Consequently, current evidence remains insufficient to establish causality or support laser therapy as a replacement for established ophthalmologic management strategies for glaucoma and other ocular complications. This narrative review evaluates current evidence regarding the effects of laser treatment for PWS on ocular manifestations in patients with SWS while emphasizing distinctions among laser modalities, limitations of the existing literature, and the importance of coordinated multidisciplinary dermatologic and ophthalmologic care.

## 2 Methods of literature search

A narrative literature review was conducted to evaluate the effects of laser treatment of port-wine stains on ocular manifestations in patients with Sturge-Weber syndrome. A literature search was performed using the PubMed and Google Scholar databases for English-language studies published through 2025 using combinations of the keywords “Sturge-Weber syndrome”, “port-wine stain”, “pulsed dye laser”, “photodynamic therapy”, “intense pulsed light”, “glaucoma”, “intraocular pressure”, and “choroidal hemangioma”. Included literature consisted of clinical trials, observational studies, retrospective cohorts, case series, case reports, and relevant review articles addressing dermatologic laser treatment and ocular outcomes in SWS. Articles unrelated to ocular manifestations and studies lacking relevance to laser-based treatment were excluded. Due to substantial heterogeneity in study design, laser modality, outcome measures, and follow-up duration, a qualitative narrative synthesis was performed rather than a formal meta-analysis.

## 3 Pathophysiology of Sturge-Weber syndrome

### 3.1 Genetic and molecular basis

The clinical manifestations of Sturge-Weber syndrome (SWS) arise from somatic mosaic mutations in *GNAQ* (9q21.2), affecting only subsets of cells and contributing to the sporadic nature of the disorder. The *GNAQ* gene encodes a 359 amino acid protein that physiologically cycles between inactive guanosine diphosphate (GDP)-bound and active guanosine triphosphate (GTP)-bound states (7). The underlying genetic defect most commonly involves a gain-of-function mutation causing an Arginine (R) to Glutamine (Q) substitution at the 183 amino acid residue (p.R183Q)1. The *GNAQ* protein contains a RAS-like GTPase domain and an α-helical domain, which regulate the GTP/GDP binding site and downstream signaling activity. Conservation of this binding sequence across species highlights its importance in maintaining physiologic vascular signaling pathways.

The p.R183Q mutation impairs GTP hydrolysis, prolonging the active state of the protein and contributing to persistent downstream signaling (7). Under physiologic conditions, conversion of active *GNAQ*-GTP to the inactive *GNAQ*-GDP form is accelerated by GTPase-activating proteins (GAPs). Additional regulatory proteins including arrestins, G protein-coupled receptor kinases (GRKs), and regulators of G protein signaling (RGS) proteins also contribute to pathway inactivation. Conversely, guanine nucleotide exchange factors (GEFs) promote replacement of GDP with GTP, favoring pathway activation (7). Collectively, disruption of these regulatory mechanisms prolongs Gαq signaling and contributes to endothelial dysfunction and abnormal vascular development.

Persistent activation of the Gαq pathway contributes to overstimulation of downstream signaling cascades including phospholipase C-β (PLCβ), resulting in increased production of inositol 1,4,5-triphosphate (IP3) and diacylglycerol (DAG) (8). These signaling abnormalities are thought to contribute to abnormal endothelial proliferation, vascular dilation, and dysregulated angiogenesis. Elevated vascular endothelial growth factor (VEGF) expression identified within SWS-associated vascular lesions further suggests secondary angiogenic stimulation that may compound the primary *GNAQ*-driven vascular abnormality (9).

### 3.2 Vascular abnormalities

The vascular abnormalities associated with SWS involve dysfunctional and dilated blood vessels affecting the skin, eyes, and leptomeninges. Shared vascular dysregulation likely contributes to the relationship between cutaneous port-wine stains and ocular manifestations including glaucoma and choroidal hemangiomas. Leptomeningeal vascular malformations contribute to neurologic manifestations including seizures, developmental delay, and cognitive impairment. Epilepsy remains one of the most significant neurologic complications in SWS, and seizures may demonstrate resistance to medical therapy in nearly 60% of affected patients (10). Persistent vascular dysregulation involving both superficial and deeper vascular structures contributes to the multisystem manifestations characteristic of SWS.

## 4 Port-wine stains: characteristics and treatment

### 4.1 Clinical presentation of PWS in SWS

Port-wine stains (PWS) are cutaneous capillary malformations that typically present as pink or red patches on the face and may progressively darken, thicken, and develop nodularity over time. Disease progression and clinical appearance vary considerably, with differences in lesion size, color, shape, vascular depth, and distribution. In atypical presentations, biopsy may occasionally be required to support diagnosis and avoid misdiagnosis or inappropriate treatment. Development of hypertrophy and nodularity within PWS has been associated with dysregulated signaling pathways involving Protein Kinase C alpha (PKCα) and phosphatidylinositol 3-kinase (11).

Clinical evaluation of PWS may be supplemented by imaging modalities including magnetic resonance imaging (MRI) and computed tomography (CT) to assess deeper vascular involvement and associated neurologic abnormalities. Dermoscopy also represents a valuable noninvasive diagnostic tool for evaluating vascular architecture within PWS (12). Dermoscopic examination may assist in differentiating PWS from other vascular lesions including granuloma telangiectaticum, hemangiomas, solitary angiokeratomas, and subungual hematomas. Reported dermoscopic findings include irregular vascular networks in approximately 88% of lesions and parallel vascular patterns in 11% of cases (13).

### 4.2 Laser treatment of PWS

Pulsed dye laser (PDL) therapy remains the standard treatment for PWS in patients with Sturge-Weber syndrome. PDL commonly utilizes wavelengths near 595 nm with short pulse durations to target superficial vascular lesions (14). The mechanism of PDL involves selective photothermolysis, in which hemoglobin within abnormal blood vessels absorbs laser energy, resulting in thermal coagulation and vascular destruction (15). Multiple treatment sessions are often required to achieve progressive lesion lightening and improvement in skin texture.

Despite its role as the standard treatment modality, complete clearance of PWS remains uncommon. Fewer than 10% of patients achieve complete clearance with PDL monotherapy, while recurrence rates ranging from 16% to 50% have been reported, potentially due to revascularization and laser-induced angiogenesis (16). Treatment response varies substantially depending on lesion location, vessel size, vascular depth, flow characteristics, hemoglobin concentration, patient age, and skin type. PWS involving the lateral face have been reported to show greater responsiveness to PDL than centrally located lesions (17, 18). Shorter treatment intervals have also been associated with improved treatment efficiency in some studies (19).

Short-term adverse effects associated with PDL therapy include transient erythema, edema, blistering, and post-inflammatory hyperpigmentation, whereas permanent scarring is relatively uncommon (17). Although PDL is generally considered safe and remains the best-established treatment for superficial PWS, recalcitrant or hypertrophic lesions may require additional therapeutic approaches.

Additional vascular-targeted modalities including neodymium-doped yttrium aluminum garnet (Nd: YAG) laser therapy, intense pulsed light (IPL), and Alexandrite laser therapy have also been explored in selected cases. These modalities differ substantially from PDL in wavelength spectrum, tissue penetration depth, vascular selectivity, and adverse effect profile. Nd: YAG laser therapy penetrates more deeply and may be useful for treatment-resistant or hypertrophic lesions; however, it is associated with increased risk of local complications including pigmentary alteration, crusting, and scarring (20). IPL utilizes broad-spectrum noncoherent light ranging from approximately 515 to 1200 nm and has demonstrated variable efficacy depending on lesion characteristics (15). However, IPL has shown limited responsiveness in some lesions involving the V2 trigeminal distribution (21). Because available studies evaluating these modalities are heterogeneous in design, treatment protocols, and outcome assessment, direct comparison between laser systems remains challenging. Consequently, selection of laser modality may be individualized based on lesion characteristics, treatment goals, patient factors, and risk profile.

## 5 Ocular manifestations in Sturge-Weber syndrome

### 5.1 Common ocular complications

Glaucoma represents one of the most significant ocular complications associated with Sturge-Weber syndrome (SWS) and is thought to result from elevated intraocular pressure (IOP) through multiple mechanisms. Glaucoma may present with a bimodal onset pattern, affecting approximately 30% to 70% of patients with SWS, with nearly 60% of cases developing during infancy and the remaining 40% occurring later in childhood or adulthood (22). One proposed mechanism contributing to elevated IOP involves capillary malformations affecting the conjunctival and episcleral vasculature which can result in elevated episcleral venous pressure (22). Increased venous pressure may impair aqueous humor outflow from the anterior chamber, contributing to progressive IOP elevation. Episcleral hemangiomas and abnormal arteriovenous vascular connections may further increase episcleral venous congestion and contribute to glaucomatous progression.

Another proposed mechanism involves developmental abnormalities of the anterior chamber (iridocorneal) angle. The iridocorneal angle, formed by the peripheral iris and peripheral cornea, contains the trabecular meshwork and Schlemm’s canal which regulate aqueous humor drainage. Gonioscopic findings in early-onset glaucoma associated with SWS include indistinct angle structures, anterior iris insertion, anterior displacement of the iris root, and poor scleral spur development, findings that resemble primary congenital glaucoma (23). Consequently, early-onset glaucoma in SWS may involve both elevated episcleral venous pressure and congenital angle abnormalities, whereas late-onset glaucoma is more commonly associated with elevated episcleral venous pressure alone (24).

Choroidal hemangiomas (CHs) represent another important ocular manifestation of SWS and may contribute to visual impairment, retinal detachment, or secondary glaucoma if not appropriately monitored and managed (25). Amblyopia may also occur secondary to eyelid hemangiomas that alter the ocular surface or interfere with normal visual axis development. Additional ocular manifestations include conjunctival vascular engorgement, ocular redness, tearing, eye enlargement, pain, strabismus, and progressive vision changes (26). Less common findings include iris heterochromia, retinal vascular tortuosity, arteriovenous vascular communications, and exudative retinal detachment (27). Choroidal thickening and associated vascular dysfunction may contribute to subretinal fluid accumulation and retinal detachment in advanced cases (28).

### 5.2 Pathophysiology of ocular involvement

The *GNAQ* mutation associated with SWS contributes to multiple vascular abnormalities involving ocular tissues and is thought to play a central role in the pathophysiology of glaucoma and other ocular manifestations. Abnormal vascular proliferation and malformed episcleral and choroidal vasculature may contribute to elevated intraocular pressure and impaired ocular drainage. The distribution of vascular malformations across trigeminal nerve dermatomes is associated with varying levels of ophthalmologic and neurologic risk. PWS involving the V1 dermatome demonstrated a 6.7% risk of glaucoma and a 26.7% risk of neurologic manifestations, whereas lesions involving the V2 dermatome demonstrated no reported glaucoma risk and a 3.1% risk of neurologic manifestations (29). Combined involvement of the V1 and V2 dermatomes was associated with a 31.8% risk of glaucoma and a 54% risk of neurologic involvement, while involvement of all three trigeminal branches was associated with an even greater risk of neurologic disease (29). Choroidal hemangiomas similarly arise from abnormal vascular proliferation within the choroid and may disrupt normal ocular structure and function through progressive vascular congestion and exudative complications.

## 6 Impact of laser treatment on ocular health

### 6.1 Review of existing literature

The effects of laser treatment for port-wine stains (PWS) on ocular manifestations in patients with Sturge-Weber syndrome (SWS) have been evaluated in a limited number of studies with variable methodologies and outcome measures. Reported ocular outcomes include changes in intraocular pressure (IOP), glaucoma progression, and choroidal hemangioma (CH) characteristics. However, the currently available evidence remains heterogeneous and consists largely of retrospective studies, observational cohorts, case series, and isolated case reports, limiting reproducibility and preventing definitive conclusions regarding causality or long-term ophthalmologic benefit.

Several studies have suggested possible associations between treatment of periocular PWS and short-term changes in ocular hemodynamics. Hemoporfin-mediated photodynamic therapy (PDT), a mechanistically distinct modality from pulsed dye laser (PDL), was associated with an average reported IOP reduction of approximately 2.13 mmHg in patients with periocular PWS30. Hemoporfin acts as a photosensitizing agent that generates reactive oxygen species following light activation, resulting in selective vascular endothelial damage and constriction of abnormal vessels. Proposed mechanisms for IOP reduction include decreased episcleral venous congestion and reduced blood flow through periocular vascular malformations (30). However, the magnitude and long-term clinical significance of this reported IOP reduction remain uncertain.

**Figure 1:**
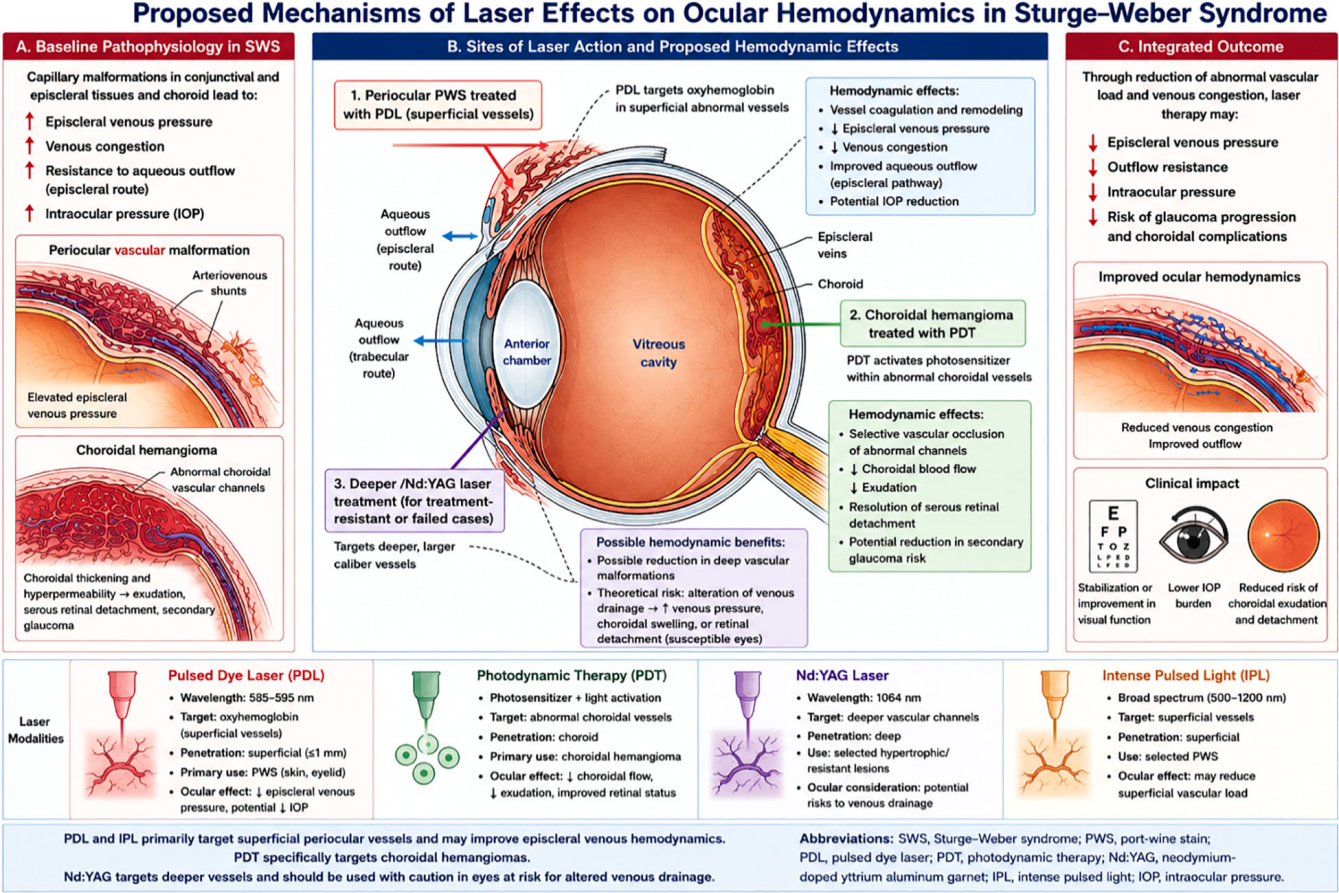
Proposed mechanisms of laser-mediated effects on ocular hemodynamics in Sturge-Weber syndrome. Illustration demonstrating proposed effects of pulsed dye laser (PDL), photodynamic therapy (PDT), and deeper vascular laser modalities on episcleral venous pressure, aqueous humor outflow, intraocular pressure, and choroidal vascular abnormalities in patients with Sturge-Weber syndrome. Proposed mechanisms remain incompletely characterized and are derived primarily from observational and mechanistic studies.

Early laser treatment of PWS may also reduce progression of hypertrophic lesions and vascular nodularity in selected patients (31). PDT has additionally demonstrated potential benefit in selected cases of choroidal hemangioma through vascular targeting and reduction of subretinal exudation. Nevertheless, the substantial variability in study design, laser modality, treatment protocols, and follow-up duration highlights the need for standardized prospective investigations. Current evidence therefore remains hypothesis-generating, and laser therapy should not be interpreted as a replacement for established ophthalmologic management strategies for glaucoma or other ocular complications (**Figure 1**; **Table 1**).

**Table 1:** Representative reported ocular effects of vascular-directed laser modalities in Sturge-Weber syndrome.

Laser Modality	Representative Reference(s)	Proposed Ocular Effect	Reported Findings	Key Limitations
Pulsed dye laser (PDL)	(32, 33)	Reduction in superficial vascular congestion and possible improvement in episcleral venous drainage	Observational reports describe modest post-treatment IOP reductions in periocular PWS, although some studies report no clinically significant IOP change	Evidence largely observational; inconsistent findings; limited long-term ophthalmologic follow-up
Photodynamic therapy (PDT)	(30, 34–37)	Selective vascular occlusion and reduction of choroidal vascular congestion	Reported reduction in IOP, decreased subretinal exudation, retinal reattachment, and improvement in visual acuity in selected cases	Small cohorts and case reports; lack of standardized controls and long-term follow-up
Nd: YAG laser	(34)	Deeper vascular targeting for hypertrophic or treatment-resistant lesions	Theoretical benefit in deeper vascular malformations with concern for altered venous drainage and ocular vascular complications	Limited ocular-specific evidence and potential risk of choroidal swelling or retinal complications
Combination and adjunctive therapies	(32, 37–39)	Enhanced vascular targeting and reduced revascularization	Adjunctive rapamycin, oxymetazoline, anti-VEGF therapy, and hemoglobin-targeting strategies may improve treatment durability and vascular selectivity	Primarily investigational with limited clinical validation and standardized outcome assessment

Reported findings are derived primarily from observational studies, retrospective cohorts, case series, and isolated case reports with heterogeneous methodologies, treatment protocols, and ophthalmologic outcome measures. The table is intended to provide a qualitative overview of representative findings discussed in the current literature and should not be interpreted as establishing causality or definitive clinical efficacy.

Summary of representative findings from currently available literature evaluating potential ocular effects associated with pulsed dye laser (PDL), photodynamic therapy (PDT), neodymium-doped yttrium aluminum garnet (Nd: YAG) laser therapy, and adjunctive vascular-targeted treatment approaches in patients with Sturge-Weber syndrome.

### 6.2 Effects of laser treatment on intraocular pressure

Laser treatment of PWS has generated interest regarding possible effects on intraocular pressure in patients with SWS, particularly in cases involving periocular lesions. However, evidence supporting a direct relationship between laser therapy and sustained IOP reduction remains limited and inconsistent. Existing studies differ substantially in laser modality, patient population, treatment location, and duration of ophthalmologic follow-up. PDL remains the standard treatment for superficial PWS and functions through selective photothermolysis targeting hemoglobin within abnormal superficial vasculature (15). Several observational reports have suggested that treatment of periocular PWS with PDL may be associated with modest reductions in IOP, potentially through decreased episcleral vascular congestion and improved venous drainage (32). Patients undergoing PDL treatment for eyelid PWS showed decreased post-treatment IOP compared with patients whose lesions did not involve the periocular region (32). However, the mechanisms underlying these observations remain incompletely understood, and not all studies have demonstrated clinically meaningful IOP reduction (33). Importantly, currently available evidence does not establish that PDL directly prevents glaucoma progression or replaces standard glaucoma therapy.

Vascular laser modalities with deeper penetration, including neodymium-doped yttrium aluminum garnet (Nd: YAG) laser therapy, have also been explored in treatment-resistant or hypertrophic lesions. These deeper vascular therapies differ significantly from PDL in tissue penetration and vascular targeting. Concerns have been raised that excessive alteration of alternative venous drainage pathways following deeper laser treatment could theoretically contribute to elevated venous pressure, choroidal swelling, or retinal detachment in susceptible patients (34). Consequently, caution is warranted when considering deeper vascular laser modalities in patients with significant ocular involvement.

PDT represents a mechanistically distinct therapy from PDL and should not be interpreted interchangeably. Unlike PDL, PDT combines a photosensitizing agent with light activation to induce selective vascular endothelial injury and vascular occlusion. PDT may exert less diffuse impact on retinal vasculature while reducing subretinal choroidal exudation (34). Comparative studies have suggested that PDT may demonstrate efficacy comparable to PDL for selected flat pink lesions in children and potentially greater efficacy in some adult lesions (35). However, available evidence remains heterogeneous and largely observational.

Although laser therapy may contribute to dermatologic improvement and possible short-term changes in ocular vascular dynamics, current evidence remains insufficient to support laser treatment as a standalone glaucoma management strategy. Early-onset glaucoma in SWS frequently involves congenital anterior chamber abnormalities that often require surgical intervention, whereas late-onset disease is more commonly managed with topical therapies that suppress aqueous humor production or increase uveoscleral outflow (9). Dermatologic response to PDL may also vary according to trigeminal nerve distribution, with V3 lesions demonstrating greater responsiveness than V2 lesions (29).

Recent advances in ophthalmologic imaging technologies, including optical coherence tomography (OCT), have improved the ability to monitor optic nerve morphology, retinal architecture, and glaucoma progression (40). OCT provides high-resolution cross-sectional imaging and three-dimensional visualization of ocular structures which allows for more precise longitudinal assessment of treatment response and disease progression (40). Integration of these imaging modalities into multidisciplinary management strategies may improve monitoring of patients with periocular vascular involvement undergoing laser therapy. Additional studies are needed to better define the long-term ocular effects and molecular consequences of laser treatment in SWS.

### 6.3 Influence on choroidal hemangiomas and other ocular features

Choroidal hemangiomas represent a major ocular manifestation of SWS and may occur in up to 71% of affected patients (27). These vascular lesions may contribute to subretinal fluid accumulation, retinal detachment, visual impairment, and secondary glaucoma if not appropriately monitored and managed. Among currently available vascular-directed therapies, PDT has demonstrated the strongest evidence for direct treatment of choroidal hemangiomas.

PDT selectively targets abnormal vascular tissue within the choroid through activation of a photosensitizing agent, resulting in vascular endothelial injury and vascular occlusion. A case report describing PDT treatment of a patient with diffuse choroidal hemangioma demonstrated resolution of subretinal fluid, retinal reattachment, and improvement in visual acuity to 20/125 over a one-year follow-up period (36). Although these findings are encouraging, they remain limited by small sample size, lack of controls, and variable follow-up duration.

PDL, in contrast, primarily targets superficial cutaneous vasculature and has not been established as a direct treatment for choroidal hemangiomas. Nevertheless, interest remains regarding whether treatment of periocular PWS could indirectly influence ocular vascular congestion or hemodynamics. Current evidence remains insufficient to establish a consistent relationship between cutaneous PDL therapy and regression of intraocular vascular lesions.

Combination therapeutic approaches involving PDL, and additional vascular-targeted therapies have also been explored in efforts to improve treatment efficacy and reduce revascularization. Experimental approaches involving hemoglobin-laden vesicles have been proposed to enhance vascular targeting and improve laser energy absorption within deeper vascular lesions (38). Adjunctive vasoconstrictive agents including oxymetazoline have also demonstrated potential to enhance vascular selectivity and treatment response (39). Additionally, antiangiogenic therapies such as rapamycin have been investigated in combination with PDL to reduce revascularization and improve treatment durability (32). Although these approaches remain investigational, they highlight ongoing interest in multimodal vascular-targeted treatment strategies for SWS-associated vascular abnormalities.

Because existing studies evaluating PDL, PDT, IPL, Nd: YAG, and combination therapies differ substantially in mechanism, penetration depth, vascular selectivity, and adverse effect profiles, findings involving these modalities should not be interpreted interchangeably. Furthermore, many available studies prioritize dermatologic improvement rather than standardized ophthalmologic endpoints. Consequently, additional prospective studies with standardized ocular outcome measures and long-term follow-up could help better define the role of laser therapy in the management of ocular manifestations associated with SWS.

## 7 Clinical implications and management strategies

### 7.1 Integrated dermatologic and ophthalmologic care

Management of Sturge-Weber syndrome requires close collaboration between dermatologists and ophthalmologists due to the overlap between cutaneous vascular malformations and ocular complications. Patients with periocular port-wine stains should undergo routine ophthalmologic evaluation, including intraocular pressure assessment and appropriate ocular imaging when indicated, given the increased risk of glaucoma and choroidal vascular abnormalities. Dermatologists performing pulsed dye laser (PDL) therapy should communicate with ophthalmologists regarding lesion distribution, treatment plans, and potential ocular concerns, particularly in patients with known periocular involvement.

Although laser therapy may contribute to dermatologic improvement and possible short-term alterations in ocular vascular dynamics, current evidence remains insufficient to support laser treatment as a substitute for standard glaucoma management. Patients with established glaucoma should continue receiving appropriate ophthalmologic monitoring and intraocular pressure-lowering therapies regardless of dermatologic treatment response. Early recognition of ocular symptoms including visual changes, ocular pain, tearing, or progressive redness remains important for timely intervention. Because treatment response and disease severity vary substantially among patients with SWS, management strategies should be individualized based on lesion characteristics, ocular findings, patient age, and overall disease burden. Coordinated multidisciplinary care may improve long-term clinical monitoring, optimize treatment selection, and enhance quality of life for patients with SWS.

## 8 Future directions and research needs

### 8.1 Gaps in current knowledge

Despite increasing use of pulsed dye laser (PDL) therapy for port-wine stains (PWS) in patients with Sturge-Weber syndrome (SWS), its long-term effects on ocular complications and underlying vascular mechanisms remain undercharacterized. Current evidence suggests possible associations between treatment of periocular vascular lesions and short-term ocular changes; however, the available literature remains heterogeneous and is composed primarily of observational studies, retrospective cohorts, and case reports. Consequently, current evidence remains insufficient to establish causality, determine durability of response, or replace established ophthalmologic medical and surgical interventions.

Interpretation of the current literature is limited by substantial variability in study design, laser modality, treatment parameters, follow-up duration, and ophthalmologic outcome assessment. Much of the available evidence consists of retrospective studies, small observational cohorts, case series, and isolated case reports which increases susceptibility to selection bias and limits generalizability of findings. Additionally, many studies prioritize dermatologic improvement rather than standardized ocular endpoints, making comparison across studies challenging. The absence of large prospective investigations and long-term ophthalmologic follow-up further limits the ability to determine durability of response or define the precise role of laser therapy in comprehensive SWS management.

Future investigations could prioritize prospective longitudinal studies with standardized ophthalmologic outcome measures, including intraocular pressure trends, glaucoma progression, retinal findings, and choroidal hemangioma outcomes. Greater consistency in reporting laser modality, treatment parameters, lesion distribution, follow-up duration, and adverse events could improve comparability across studies and strengthen reproducibility of findings. Additional mechanistic investigations are also needed to clarify how vascular-targeted therapies may influence ocular hemodynamics, episcleral venous pressure, and vascular remodeling in SWS.

### 8.2 Innovative treatment approaches

Emerging combination therapeutic strategies involving laser therapy and antiangiogenic agents have demonstrated potential to improve treatment durability and reduce revascularization in PWS management (31). Adjunctive therapies targeting angiogenic signaling pathways may help address persistent vascular dysregulation associated with SWS. Circumscribed choroidal hemangiomas have been reported to respond to photodynamic therapy (PDT) combined with anti-vascular endothelial growth factor (anti-VEGF) therapy, with reported improvement in subretinal fluid accumulation and visual acuity in selected cases (37). However, available evidence remains limited and largely observational.

Advances in molecular profiling and targeted vascular therapies may further support the development of individualized treatment strategies for patients with SWS. Continued investigation of pharmacologic agents targeting abnormal vascular signaling pathways may expand future therapeutic options (33). Nevertheless, additional prospective studies with long-term follow-up and standardized clinical endpoints remain necessary before these emerging approaches can be broadly incorporated into routine clinical practice.

## 9 Conclusion

Laser treatment of port-wine stains in Sturge-Weber syndrome may have implications beyond cosmetic improvement and could potentially influence ocular vascular dynamics, particularly in patients with periocular involvement. Current evidence supports the importance of multidisciplinary management involving coordinated dermatologic treatment and longitudinal ophthalmologic surveillance. However, available studies remain heterogeneous and consist predominantly of small observational studies, retrospective cohorts, and case reports with limited long-term follow-up. Although preliminary findings suggest possible associations between vascular-directed laser therapy and changes in intraocular pressure or choroidal vascular abnormalities, current evidence remains insufficient to establish causality or support laser therapy as a replacement for established glaucoma management strategies. Additional prospective studies integrating standardized ophthalmologic outcomes, mechanistic investigations, and long-term follow-up are necessary to better define the role of laser therapy within comprehensive SWS management.
